# Determinants of Inadequate Minimum Dietary Diversity Intake Among Children Aged 6–23 Months in Sub-Saharan Africa: Pooled Prevalence and Multilevel Analysis of Demographic and Health Survey in 33 Sub-Saharan African Countries

**DOI:** 10.3389/fnut.2022.894552

**Published:** 2022-07-01

**Authors:** Daniel Gashaneh Belay, Fantu Mamo Aragaw, Rediet Eristu Teklu, Samrawit Mihret Fetene, Wubshet Debebe Negash, Desale Bihonegn Asmamaw, Elsa Awoke Fentie, Tewodros Getaneh Alemu, Habitu Birhan Eshetu, Ever Siyoum Shewarega

**Affiliations:** ^1^Department of Human Anatomy, College of Medicine and Health Sciences, University of Gondar, Gondar, Ethiopia; ^2^Department of Epidemiology and Biostatistics, Institute of Public Health, College of Medicine and Health Sciences, University of Gondar, Gondar, Ethiopia; ^3^Department of Health Systems and Policy, Institute of Public Health, College of Medicine and Health Sciences, University of Gondar, Gondar, Ethiopia; ^4^Department of Reproductive Health, Institute of Public Health, College of Medicine and Health Sciences, University of Gondar, Gondar, Ethiopia; ^5^Department of Pediatrics and Child Health Nursing, School of Nursing, College of Medicine and Health Sciences, University of Gondar, Gondar, Ethiopia; ^6^Department of Health Education and Behavioral Sciences, Institute of Public Health, College of Medicine and Health Sciences, University of Gondar, Gondar, Ethiopia

**Keywords:** inadequate minimum dietary diversity, children, Ethiopia, multilevel, nutrition

## Abstract

**Background:**

Inappropriate feeding practices result in significant threats to child health by impaired cognitive development, compromised educational achievement, and low economic productivity, which becomes difficult to reverse later in life. There is minimal evidence that shows the burden and determining factors of inadequate dietary intake among children aged under 2 years in sub-Saharan African (SSA) countries. Therefore, this study aimed to assess the pooled magnitude, wealth-related inequalities, and other determinants of inadequate minimum dietary diversity (MDD) intake among children aged 6**–**23 months in the SSA countries using the recent 2010–2020 DHS data.

**Methods:**

A total of 77,887 weighted samples from Demographic and Health Survey datasets of the SSA countries were used for this study. The Microsoft Excel and STATA version 16 software were used to clean, extract, and analyze the data. A multilevel binary logistic regression model was fitted. The concentration index and curve were applied to examine wealth-related inequalities in the outcomes. *P*-value < 0.05 with 95% CI was taken to declare statistical significance.

**Results:**

The pooled magnitude of inadequate MDD intake among children aged 6–23 months in SSA was 76.53% (95% CI: 73.37, 79.70), ranging from 50.5% in South Africa to 94.40% in Burkina Faso. Individual-level factors such as women having secondary and above education (AOR = 0.66; 95% CI; 0.62, 0.70), being employed (AOR = 0.76; 95% CI; 0.72, 0.79), having household media exposure (AOR = 0.69; 95% CI; 0.66, 0.72), richest wealth (AOR = 0.46; 95% CI; 0.43, 0.50), having health institution delivery (AOR = 0.87;95% CI; 0.83, 0.91), and community-level factor such as living in upper middle-income country (AOR = 0.42; 95% CI; 0.38, 0.46) had a significant protective association, whereas rural residence (AOR = 1.29; 95% CI; 1.23, 1.36) has a significant positive association with inadequate MDD intake among children aged 6–23 months. Inadequate MDD intake among children aged 6–23 months in SSA was disproportionately concentrated on the poor households (pro-poor) (C = −0.24; 95% CI: −0.22, −0.0.26).

**Conclusion and Recommendations:**

There is a high magnitude of inadequate minimum dietary diversity intake among children aged 6–23 months in SSA. Variables such as secondary and above maternal education, having an employed mother, having exposure to media, richest wealth, having health institution delivery, and living in the upper middle-income country have a significant negative association, whereas living in rural residence has a significant positive association with inadequate MDD intake. These findings highlight that to increase the MDD intake in the region, policy makers and other stakeholders need to give prior attention to enhancing household wealth status, empowering women, and media exposure.

## Introduction

Malnutrition has a significant impact on the health of a person’s life course and is closely tied to cognitive and social development, particularly in early childhood (1, 2). Globally, inadequate child feeding practices and their consequences remain a major hindrance to poverty reduction and sustainable socioeconomic development (3). In children aged 6 months and above, complementary food needs to be started in order to supplement the breastfeeding, which becomes inadequate as children grow older (1). To overcome these problems, in children who aged 6 months of age and above, complementary feeding needs to be started (2).

Minimum dietary diversity is one of the eight indicators of infant and young child feeding (IYCF) practices launched by the World Health Organization (WHO) (4, 5). It is a proxy for adequate micronutrient density of foods and is defined as children who received four or more from the following seven food groups (5, 6). These food groups are grains, roots and tubers, legumes and nuts, dairy products (milk, yogurt, and cheese), flesh foods (meat, fish, poultry, and liver/organ meats), eggs, vitamin-A rich fruits and vegetables, and other fruits and vegetables (2, 7, 8). This means that the child had a high likelihood of consuming at least one animal-source food and at least one fruit or vegetable in a day, in addition to a staple food (grain, root, or tuber) (4, 5).

Suboptimal IYCF practices remain serious public health concerns in children, especially in resource-poor communities (9). The first 1,000 days (time from conception up to 2 years of age) of the child’s life provide a critical window of opportunity to ensure survival, growth, and development through optimum IYCF practices (10). Inappropriate IYCF practices during this period result in significant threats to child’s health by impairing cognitive development, compromising educational achievement, and lowering economic productivity, which becomes difficult to reverse later in life (8, 10, 11). More than two-thirds of malnutrition-related child deaths are associated with inappropriate feeding practices during the first 2 years of life (12). Undernutrition is linked to just under half of all the deaths of children aged under five each year (10, 13). Nearly one-third of child deaths could be prevented by optimal complementary feeding practices (14). Research has shown that in sub-Saharan Africa (SSA), children lost up to 2.5 years of schooling if there was a famine, while they were *in utero* and during their childhood (13).

Global estimates for feeding children aged 6–23 months indicate substantial room for improvement (10, 15). In many countries, less than one-quarter of children are reported not getting the nutrition they need to grow well, particularly in the crucial first 1,000 days (13, 16). The magnitude of inadequate minimum dietary diversity (MDD) in Nepal was 53.5% (17); in India, it ranges from 36.15 to 77% (18, 19), MDD in Indonesia was 46.7% (20), and MDD in Sri Lanka was 29% (21).

Sociodemographic factors such as maternal education (9, 22, 23), occupation of mothers (9, 24, 25), and wealth status of the households (22, 23), whereas community-level factors such as residence (9, 22, 24, 26) and having regional variation (26–28) have a significant association with dietary diversity usage among children aged 6–23 months.

There is minimal evidence of the burden and determining factors of inadequate dietary intake among under 2 years old children in the SSA countries. Moreover, the regional variations in accessibilities and cultures of food items make it difficult to obtain the pooled estimate and to make regional comparisons. Therefore, this study used DHS data collected from 33 SSA countries in a similar design and using standardized parameters. This makes it easy to assess the pooled magnitude and factors associated with inadequate MDD intake among children aged 6–23 months.

## Materials and Methods

### Study Design, Setting, and Period

Recently collected standard cross-sectional DHS data set of the SSA countries within 10 years (2010–2020) were used as our data source. To obtain a representative sample of the recent standard DHS data from each region of SSA, 10 years of DHS data (starting from 2010) were taken for analysis. The standard DHS data set was used for each country to get all parameters and a large sample size, which can be representative of the source of population and have a particular advantage in a pooled analysis (7).

The sub-Saharan is the area in the continent of Africa that lies south of the Sahara and consists of four vast and distinct regions, i.e., Eastern Africa, Central Africa, Western Africa, and Southern Africa. A total of thirty-three SSA countries were represented in this study in the four regions, namely, Eastern Africa (Burundi, Comoros, Ethiopia, Kenya, Malawi, Mozambique, Rwanda, Tanzania, Uganda, Zambia, Zimbabwe), Southern Africa (Lesotho, Namibia, South Africa), Central Africa (Angola, Cameroon, Chad, the Democratic Republic of the Congo, Republic of the Congo, Gabon), and Western Africa (Benin, Burkina Faso, Ivory Coast, Gambia, Ghana, Guinea, Liberia, Mali, Niger, Nigeria, Senegal, Sierra Leone, Togo) (29) (Figure 2). Together, they constitute an area of 9.4 million square miles and a total population of 1.1 billion inhabitants (30). The datasets are publicly available from the DHS website www.dhsprogram.com (29). DHS collects data that are comparable across countries. The surveys are nationally representative of each country and population-based with large sample sizes. All surveys use a multistage cluster sampling method (7).

**FIGURE 1:**
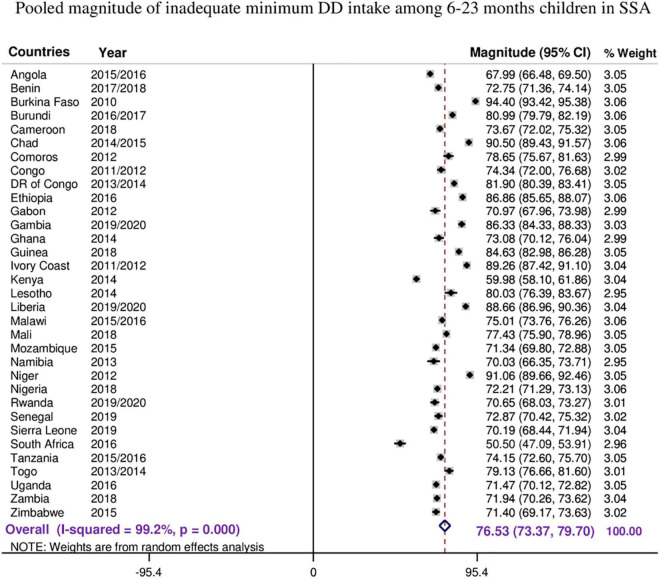
The forest plot showed that the pooled magnitude of inadequate minimum dietary diversity intake among children aged 6–23 months.

**FIGURE 2:**
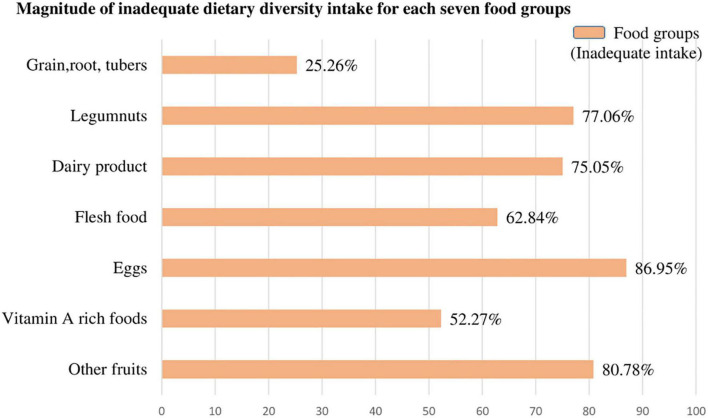
The magnitude of inadequate dietary intake for each seven food groups among children aged 6–23 months in SSA.

### Populations

All children aged 6–23 months preceding 5 years of the survey period across 33 SSA countries were our source population, whereas children aged 6–23 months in the selected Enumeration Areas (EAs) or primary sampling units of the survey clusters were our study populations. The mother or the caregiver was interviewed for the survey in each country and mothers who had more than one child within the 2 years preceding the survey were asked questions about the most recent child (6). Moreover, from the data set of included countries, children in the age category of 6–23 months, which is not assessed for MDD based on the DHS guideline, and having the missing value of the outcome variable, were excluded.

### Sample Size Determination and Sampling Method

Of the total of 47 countries located in SSA, only 41 countries had Demographic and Health Survey Report. Of these, five countries, namely, Central Africa Republic (DHS report 1994/95), Eswatini (DHS report 2006/07), Sao Tome Principe (DHS report 2008/09), Madagascar (DHS report 2008/09), and Sudan (DHS report 1989-90) have a survey report before the 2010 survey year and were excluded from further analysis. Moreover, three countries (Botswana, Mauritania, and Eritrea) were excluded due to the DHS dataset not being publicly available. Finally, a total of 33 SSA countries were included in this study.

In general, the most recent standard census frame was used in all of the surveys conducted in the selected countries. Typically, DHS samples are stratified by administrative geographic region and by urban/rural areas within each region. DHS sample designs are usually two-stage probability samples drawn from an existing sample frame. In the first stage of sampling, EAs were selected with probability proportional to size within each stratum. In selected EAs, a fixed number of households is selected by the systematic sampling method in the second stage of sampling. Finally, a fixed number of households is selected by equal probability systematic sampling in the selected cluster. The detailed sampling procedure was available in the Measure DHS program (7).

The children’s records or kid’s records (KR) DHS datasets were used. Weighted values were used before using the DHS dataset to restore the representativeness of the sample data, as the overall probability of selection of each household is not constant. DHS guidelines set four sampling weighting methods, and from that, we used the individual weight for women (v005), which is the household weight (hv005) multiplied by the inverse of the individual response rate for women in the stratum. Individual sample weights are generated by dividing (v005) by 1,000,000 before using it to approximate the number of cases (31). From 81,123 total eligible households, 78,483 had complete data for inadequate MDD intake with a response rate of 95.5%. Finally, a total weighted sample of 77,887 children in the age category of 6–23 months from all 33 countries was included in this study (Table 1).

**TABLE 1:** Sample size determination of inadequate minimum dietary diversity intake and factors associated with it among children aged 6–23 months in each sub-Saharan Africa country: based on 2010–2020 DHS.

Sub-Saharan Africa Countries with Recent DHS report from 2010/11 to 2019/20					
Regions	Countries	Standard	inadequate MDD sample size (*n*)		
		DHS year	Un weighted	Weighted	Percentage (%)
East Africa countries	Burundi	2016/17	3,958	4,078	5.24
	Comoros	2012	727	728	0.94
	Ethiopia	2016	2,806	2,983	3.83
	Kenya	2014	2,822	2,610	3.35
	Malawi	2015/16	4,594	4,611	5.92
	Mozambique	2015	3,158	3,312	4.25
	Rwanda	2019/2020	1,133	1,159	1.49
	Tanzania	2015/16	3,124	3,071	3.94
	Uganda	2016	4,344	4,282	5.5
	Zambia	2018	2,825	2,756	1.06
	Zimbabwe	2015	1,531	1,584	3.54
	Subtotal		31,022	31,175	40.03
	Angola	2015/16	3,962	3,659	4.7
	Cameroon	2018	2,635	2,739	3.52
Central Africa countries	Chad	2014/15	2,791	2,878	3.69
	DR Congo	2013/14	2,572	2,495	3.2
	Congo	2011/12	1,063	1,339	1.72
	Gabon	2012	1,122	875	1.12
	Subtotal		14,586	13,984	17.95
	Benin	2017/18	3,913	3,917	5.03
	Burkina Faso	2011	2,080	2,100	2.7
	Ivory Coast	2011/12	1,095	1,090	1.4
	Gambia	2019/20	1,160	1,134	1.46
	Ghana	2014	879	864	1.11
West Africa countries	Guinea	2018	1,892	1,846	2.37
	Liberia	2019/20	1,513	1,342	1.72
	Mali	2018	2,723	2,872	3.69
	Niger	2012	1,523	1,588	2.04
	Nigeria	2018	9,084	9,167	11.77
	Senegal	2019	1,349	1,262	1.62
	Sierra Leone	2019	2,648	2,626	3.37
	Togo	2013/14	1,063	1,037	1.33
	Subtotal		30,922	30,842	39.60
Southern Africa countries	Lesotho	2014	468	463	3.37
	Namibia	2013	644	596	0.76
	South Africa	2016	841	825	2.03
	Subtotal		1,953	1,885	2.42
Total sample size			78,483	77,887	100%

### Study Variables

#### Dependent Variables

The outcome variable of this study was inadequate intake of MDD among children aged 6–23 months. During the survey, their mother was asked questions about the types of food the child had consumed during the day or night before the interview (7). If a child has not taken four out of seven food groups fed during the day or night preceding the survey, the following food items are considered as getting inadequate MDD. These items are grains, roots and tubers, legumes and nuts, dairy products (milk, yogurt, and cheese), flesh foods (meat, fish, poultry, and liver/organ meats), eggs, vitamin A rich fruits and vegetables, and other fruits and vegetables. The data of the above variables were collected similarly across all the SSA countries (5, 7) (Table 2).

**TABLE 2:** Food groups were used to assess the outcome variables (inadequate minimum dietary diversity intake) among children aged 6–23 months within SSA.

Minimum dietary diversity assessing food items	Inadequate minimum dietary diversity intake
1. Did the child take grains, roots, and tubers?	If a child is not taken four and above foods out of seven food items during the day or night preceding the survey.
2. Did the child take legumes and nuts?	
3. Did the child take dairy products (milk, yogurt, and cheese)?	
4. Did the child take flesh foods (meat, fish, poultry, and liver/organ meats)?	
5. Did the child take eggs?	
6. Did the child take vitamin A-rich fruits and vegetables?	
7. Did the child take other fruits?	

### Independent Variables

Individual and community-level independent variables have been studied. The individual-level factors included sociodemographic characteristics such as the age of the mother, mother’s employment, marital status, family size, maternal education, and household wealth status. Child-related factors such as the age of the child, sex of the child, birth order, the plurality of birth, and breastfeeding status of the child are all taken into account.

Behavioral characteristics like media exposure were studied. Media exposure status was created from the frequency of watching TV, listening to the radio, and reading a newspaper or a magazine. If a woman has at least one yes, she has considered media exposure. Health service utilization-related factors such as the place of delivery and antenatal care (ANC) visit were also considered.

The community-level factors included the place of residence, region in SSA, survey year, income level of the country, community-level media exposure, and community-level women’s education (Table 3).

**TABLE 3:** Individual and community-level independent variables in the study of inadequate minimum dietary diversity intake and associated factors among children aged 6–23 months in SSA.

Level	Variables	Measurements
Individual level variables	Age	The age of the mother/caregiver is categorized as 15–19, 20–35, and 36–49.
	Sex	Sex of the household head as male or female.
	Education level	Educational attainment is categorized as uneducated, primary, secondary, and above educational status.
	Marital status	The marital status of the mothers is categorized as married or not married.
	Occupation of women	The occupation of women is categorized as working and not working.
	Family size	Categorized as 1–4, 510, and 11 and above.
	Media exposure	A composite variable was obtained by combining whether a respondent reads newspaper/magazine, listens to the radio, and watches television with a value of “0” if women were not exposed to at least one of the three media, and “1” if a woman has access/exposure to at least one of the three media (32).
	Wealth index	The datasets contained a wealth index that was created using principal components analysis coded as poorest, poorer, middle, richer, and richest in the DHS data set. For this study, we recorded it in three categories poor (including poorer and poorest), middle and rich (includes richer and richest).
	Birth order	Categorized as birth order less than or equal to three and above three.
	Sex of the child	The sex of the child is categorized as male or female.
	Age of the child	The age of the child is categorized as 6–8, 9–11, and 12–23 months.
	Plurality of birth	Categorized as single birth or more than one birth.
	Currently, breastfeed	Categorized as breastfeeding or not breastfeeding.
	Wanted pregnancy	Categorized as wanted pregnancy or unwanted pregnancy
	Place of delivery	Classified as home delivery and health institution delivery
	ANC visit	Grouped as At least one ANC visit or not have ANC visit
Community level variables	Residency	Urban or rural based on where the household lives.
	Region in SSA	The region in sub-Saharan African region is categorized as Eastern Africa, Central Africa, Western Africa, and Southern Africa.
	Countries income level	The countries’ income status was categorized as low income, lower middle income, and upper-middle-income country based on the World Bank List of Economies classification since 2019 (33). World Bank calculated country income based on Gross National Income (GNI) per capita, which categorized as low income $1,025 or less; lower middle income, $1,026–3,995, upper middle income $3,996–12,375, and high income $12,375 or more (33).
	DHS survey year	Survey year means the recent standard DHS data collection period of each country from 2010 to 2020. Categorized as DHS year 2010–2012, 2013–2015, and 2016–2020.
	Community level poverty	The level of poverty in the community was determined by the proportion of households in the poorest and poorer quintiles obtained from the wealth index results. Categorized as low if the proportion of household which is from households belonging to the categories of poor was less than 50% and categorized as high if the proportion was greater than 50% (34, 35).
	Community level media exposure	Community-level media exposure was assessed by the proportion of women who had at least been exposed to one media, television, radio, or newspaper. It was coded as “0” for low (communities in which < 50% of women had media exposure at least for one media), “1” for high community-level media exposure (communities in which ≥ 50% of women had at least for one media (34, 35).

### Data Processing and Analysis

This study was performed based on the DHS data obtained from the official DHS measure website www.measuredhs.com after permission has been obtained *via* an online request by specifying the objectives. Standard DHS datasets were downloaded and then cleaned, integrated, transformed, and appended to produce favorable variables for the analysis. The Microsoft Excel and STATA 16 software were used to generate both descriptive and analytic statistics of the data of appended 33 countries to describe variables in the study using statistical measurements. The pooled estimate of inadequate MDD intake among children in SSA and sub-group analysis was estimated using a *metan* STATA command.

### Model Building for Multilevel Analysis

The DHS data have a hierarchical nature, and children aged 6–23 months and mothers were nested within a cluster. This might violate the standard logistic regression model assumptions such as the independence and equal variance assumptions. Therefore, a multilevel binary logistic regression model was fitted. Four models were fitted for multilevel analysis. The model without exposure variables (null model) was used to check the variability of inadequate minimum DD intake across the cluster. The association of individual-level variables with the outcome variable (Model I) and the association of community-level variables with the outcome variable (Model II) were assessed. In the final model (Model III), the association of both individual and community-level variables was fitted simultaneously with the prevalence of inadequate minimum DD intake.

Random effects or measures of variation of the outcome variables were estimated by the median odds ratio (MOR), intraclass correlation coefficient (ICC), and proportional change in variance (PCV). Taking clusters as a random variable, the MOR is defined as the median value of the odds ratio between the area at the highest risk and the area at the lowest risk area when randomly picking out two clusters: MOR=e0.95V⁢A. In contrast, the ICC reveals the variation of inadequate minimum DD between clusters is calculated as$I\,C\,C=\frac{V\,A}{V\,A+3.29}*100\%$. Moreover, the PCV reveals the variation in the prevalence of inadequate minimum DD intake among children aged 6–23 months explained by factors and calculated as $P\,C\,V=\frac{V\,n\,u\,l\,l-V\,A}{V\,n\,u\,l\,l}*100\%,$ where Vnull = variance of the initial model and VA = area/cluster level variance (35–37).

The fixed effects or measure of association was used to estimate the association between the likelihood of prevalence of inadequate minimum DD intake and individual and community-level explanatory variables. It was assessed and the strength was presented using adjusted odds ratio (AOR) and 95% confidence intervals with a *p*-value of < 0.05.

$$L\,o\,g\,\left(\frac{\pi \,i\,j}{1-\pi \,i\,j}\right)\,\beta \,o+\beta \,1\,x\,i\,j+\beta \,2\,x\,i\,j+\ldots \,u\,j+e\,i\,j$$

where π*ij* is the probability of inadequate minimum DD intake use and 1−π*ij* is the probability of adequate minimum DD intake use. ß0 is the intercept that is the effect of feeding inadequate minimum DD when the effect of all explanatory variables is absent. β1*xij* are individual and community-level variables for the i*^th^* individual in group j, respectively. The ß’s are fixed coefficients indicating that a unit increase in X can cause a ß unit increase in probability feeding inadequate minimum DD. The uj shows the random effect (effect of the community/clusters on the mother’s decision to provide inadequate minimum DD) for the j*^th^* clusters (35, 37, 38).

Model comparisons were done using the deviance test and log likelihood test and the model with the highest log-likelihood ratio and the lowest deviance was selected as the best-fitted model. The variance inflation factor (VIF) was used to detect multicollinearity, and all variables had VIF values less than 10 and the mean VIF value of the final model was 1.54.

### Concentration Curve and Index

The concentration index and concentration graph approach are used to examine socioeconomic inequalities in inadequate minimum DD intake (39, 40). The concentration curve is used to plot the cumulative percentage of the health variable (y-axis) against the cumulative percentage of the population ranked by living standards beginning with the poorest and ending with the richest (x-axis) (41). The concentration index is used to quantify and compare the degree of socioeconomic-related inequality in inadequate minimum DD intake (42, 43). The concentration index is twice the area between the concentration curve and the diagonal line and ranges from –1 to + 1. The sign of the concentration index indicates the direction of the relationship between the health variable (inadequate minimum DD) and the distribution of living standards (wealth status) (40, 41), whereas the magnitude of the concentration index reflects both the strength of the relationship and the degree of variability in the health variable (41, 44).

## Results

### Sociodemographic Characteristics of Mothers or Caregivers and the Children

A total weighted sample of 77,887 children aged 6–23 months were included in this study. Three-fourths (75.77%) of mothers/caregivers were found in the age group of 20–35 years, with a median age of 27 (IQR: 22, 33) years. More than three-fifths of women (62.26%) had formal education. Most (70.52%) of the mothers were not working. Moreover, 67.01% of children were delivered at the health facility.

Almost equal proportions of male (50.46%) and female (49.54%) children were studied. Nearly two-thirds (65.25%) of the children were found in the age group from 12 to 23 months with a median age of 14 (IQR: 10, 18) months. Almost all (96.94%) the newborn children were singleton. From the community-level variables, most (65.36%) of the respondents were rural inhabitants of whom 80.71% had inadequate MDD intake. Notably, 64% of the SSA countries included in the study were lower-income countries (Table 4).

**TABLE 4:** Sociodemographic characteristics of the mothers/caregivers and the children in a study of inadequate minimum dietary diversity intake and associated factors among children aged 6–23 months in sub-Saharan Africa: based on 2010–2020 DHS.

Variables	Categories	Inadequate dietary diversity frequency (*n* = 77,887)		Total frequency (%)
		Yes (76.53 %)	No (23.47%)	
**Sociodemographic characteristics and health service utilization of the mothers**				
Age of women (years)	15–19	5,704 (78.61)	1,552 (21.39)	7,256 (9.32)
	20–35	44,626 (75.62)	14,390 (24.38)	59,016 (75.77)
	36–49	8,807 (75.82)	2,808 (24.18)	11,616 (14.91)
Sex of household head	Male	29,836 (75.91)	9,467 (24.09)	39,303 (50.46)
	Female	29,301 (75.94)	9,283 (24.06)	38,584 (49.54)
Educational attainment of women	No education	24,586 (83.65)	4,804 (16.35)	29,391 (37.73)
	Primary education	20,570 (77.17)	6,084 (22.83)	26,654 (34.22)
	Secondary and above	13,980 (64.01)	7,862 (35.99)	21,843 (28.04)
Occupation of women	Worked	17,203 (77.96)	4,864 (22.04)	22,067 (29.48)
	Not working	39,554 (74.94)	13,227 (25.06)	52,780 (70.52)
Marital status of the mother	Married	41,714 (76.1)	13,104 (23.9)	54,818 (70.38)
	Not married	17,423 (75.52)	5,646 (24.48)	23,069 (29.62)
House hold family size	1–4	16,359 (74.67)	5,550 (25.33)	21,909 (28.13)
	5–10	35,285 (76.01)	11,136 (23.99)	46,421 (59.6)
	≥11	7,493 (78.4)	2,065 (21.6)	9,557 (12.27)
Media exposure	No	24,284 (84.04)	4,611 (15.96)	28,895 (37.14)
	Yes	34,772 (71.11)	14,125 (28.89)	48,898 (62.86)
Wealth index	Poorest	15,015 (84.25)	2,808 (15.75)	17,823 (22.88)
	Poorer	13,871 (81.45)	3,159 (18.55)	17,030 (21.86)
	Middle	12,286 (77.55)	3,557 (22.45)	15,843 (20.34)
	Richer	10,393 (71.67)	4,108 (28.33)	14,501 (18.62)
	Richest	7,571 (59.66)	5,118 (40.34)	12,689 (16.29)
Unplanned pregnancy	No	42,110 (76.17)	13,178 (23.83)	55,288 (71)
	Yes	17,015 (75.34)	5,570 (24.66)	22,585 (29.00)
Place of delivery	Home delivery	21,168 (82.38)	4,528 (17.62)	25,696 (32.99)
	Health facilities	37,967 (72.75)	14,222 (27.25)	52,189 (67.01)
ANC visits	No ANC	9,697 (82.48)	2,059 (17.52)	11,757 (15.09)
	At least one ANC	49,440 (74.76)	16,691 (25.24)	66,131 (84.91)
**Child related characteristics**				
Sex of child	Male	29,836 (75.91)	9,467 (24.09)	39,303 (50.46)
	Female	29,301 (75.94)	9,283 (24.06)	38,584 (49.54)
Age of child	6–8 months	12,440 (88.51)	1,614 (11.49)	14,055 (18.05)
	9–11 months	10,386 (79.83)	2,624 (20.17)	13,010 (16.70)
	12–23 months	36,310 (71.45)	14,512 (28.55)	50,823 (65.25)
Birth order	<three	32,662 (73.71)	11,650 (26.29)	44,313 (56.89)
	>three	26,475 (78.85)	7,100 (21.15)	33,575 (43.11)
Plurality	Single	57,372 (75.99)	18,132 (24.01)	75,505 (96.94)
	Multiple	1,764 (74.06)	618 (25.94)	2,382 (3.06)
Breast feeding status	Not breastfed	9,948 (62.64)	5,933 (37.36)	15,881 (20.39)
	breastfed	49,189 (79.33)	12,818 (20.67)	62,006 (79.61)
**Community level variables**				
Residence	Urban	15,949 (65.42)	8,431 (34.58)	24,380 (31.3)
	Rural	43,188 (80.71)	10,320 (19.29)	53,507 (68.7)
Region in SSA	Central Africa	10,769 (77.01)	3,215 (22.99)	13,984 (17.95)
	East Africa	23,124 (74.18)	8,051 (25.82)	31,175 (40.03)
	West Africa	24,038 (77.94)	6,805 (22.06)	30,843 (39.6)
	South Africa	1,205 (63.94)	680 (36.06)	1,885 (2.42)
Country income level	Lower	40,038 (78.66)	10,865 (21.34)	50,903 (65.36)
	Lower middle	15,156 (72.07)	5,873 (27.93)	21,029 (27)
	Upper middle	3,943 (66.21)	2,012 (33.79)	5,955 (7.64)
Survey year	2010–2012	9,871 (80.31)	2,421 (19.69)	12,292 (15.78)
	2013–2015	19,606 (74.94)	6,555 (25.06)	26,161 (33.59)
	2015–2019	29,660 (75.21)	9,775 (24.79)	39,434 (50.63)
Community media exposure	Low	30,253 (79.06)	8,015 (20.94)	38,267 (49.13)
	High	28,884 (72.9)	10,736 (27.1)	39,620 (50.87)
Community level women’s education	Low	30,310 (79.15)	7,983 (20.85)	38,293 (49.16)
	High	28,826 (72.8)	10,768 (27.2)	39,594 (50.84)

### The Pooled Magnitude of Inadequate Minimum Dietary Diversity Intake Among Children Aged 6–23 Months

The overall pooled estimate of inadequate MDD intake among children aged 6–23 months in the SSA countries was 76.53% (95% CI: 73.37–79.70), with *I*^2^ = 99.2% and ranging from 50.5% in South Africa to 94.40% in Burkina Faso (Figure 1).

As the *I*^2^ value was large, which shows the true variabilities (the variability not by chance) of inadequate MDD intake among 33 countries, further subgroup analyses were performed to treat this heterogeneity effect based on the region in SSA, level of income of the country, and the DHS survey year. Based on the subgroup analysis using regions in SSA, the pooled magnitude of inadequate intake of MDD ranges from 66.84% (95% CI: 49.51–84.17) in Southern Africa across three countries to 80.94% (95% CI: 75.66–86.22) among 13 West Africa countries.

Moreover, the pooled magnitude of inadequate intake of MDD across country income levels was determined. Among 21 low-income level countries, the pooled estimate of inadequate MDD intake was 76.53% (95% CI: 73.37–79.70), whereas it was 64.91% (95% CI: 56.99–72.83) across four upper middle-income countries. In addition, the pooled estimate of inadequate MDD intake in 15 countries whose DHS survey conducted before and in 2015 was 78.44% (95% CI: 72.88–84.00), whereas it was 74.97% (95% CI: 71.77–78.18**)** in 18 countries whose DHS survey was conducted after 2015 (Table 5).

**TABLE 5:** Subgroup analyses of inadequate minimum dietary diversity intake among children in SSA.

Subgroup	Categories	Number of countries	Prevalence (%) (95%CI)	I-squared	*p*-value
Region in SSA	Central Africa	6	76.59 (68.48, 84.70)	99.3%	<0.001
	Western	13	80.94 (75.66, 86.22)	99.3%	<0.001
	Eastern	11	73.87 (69.61, 78.13)	98.7%	<0.001
	Southern	3	66.84 (49.51, 84.17)	98.6%	<0.001
Income Status of the countries	Lower income	21	79.56 (75.93, 83.19)	99.2%	<0.001
	Lower middle income	8	74.28 (68.91, 79.66)	98.6%	<0.001
	Upper middle income	4	64.91 (56.99, 72.83)	97.0%	<0.001
DHS released year	Released before and in 2015	15	78.44 (72.88, 84.00)	99.3%	<0.001
	Released after 2015	18	74.97 (71.77, 78.18)	98.7%	<0.001
Total		33	76.53 (73.37, 79.70)	99.2%	<0.001

The magnitude of inadequate dietary intake for each seven food groups was present in the following figures. Most of the children (86.95%) and four of five children (80.78%) children aged 6–23 months in SSA cannot access eggs and other fruits, respectively (other than vitamin A rich fruits listed in the DHS guidelines). But children aged 6–23 months in SSA have better access to grains, roots, and tuber food groups (74.74%) (Figure 2).

### Multilevel Analysis of Factors Associated With Inadequate Minimum Dietary Diversity Intake Among Children Aged 6–23 Months in SSA

In the multilevel analysis, the random effect analyses ICC, PCV, and MOR were assessed. The ICC in the null model indicates that approximately 16% of the variations in inadequate MDD intake among children aged 6–23 months were attributed to cluster differences. The MOR value of 2.05, in the null model, also revealed that the odds of inadequate MDD among study participants were two times different between lower and higher risk clusters. Furthermore, the PCV valve in the final model indicates that about 16% of the variation in inadequate MDD intake among study participants was explained by the final model (model four). As the model is nested, log-likelihood and deviance tests were used for model comparison. The model with the highest log-likelihood and the lowest deviance was the better-fitted model, which was model four (72,558) and selected for interpretation (Table 6).

**TABLE 6:** Multilevel analysis of factors associated with inappropriate dietary diversity intake among children aged 6–23 months in sub-Saharan Africa: based on 2010 to 2020 DHS.

Variables	Categories	Null model	Model I	Model II		Model III
			AOR (95% CI)	AOR (95% CI)		AOR (95% CI)
Age of women (years)	15–19	− − − −	1.00	_—————_		1.00
	20–35	− − − −	1.04 (0.96, 1.11)	_—————_		1.07 (0.99, 1.15)
	36–49	− − − −	0.95 (0.87, 1.04)	_—————_		1.02 (0.93, 1.12)
Sex of household head	Male	− − − −	1.00	_—————_		1.00
	Female	− − − −	1.02 (0.97, 1.07)	_—————_		1.04 (0.99, 1.09)
Educational attainment of women	No education	− − − −	1.00	_—————_		1.00
	Primary education	− − − −	0.80 (0.76, 0.84)***	_—————_		0.92 (0.86, 0.96)**
	Secondary and above	− − − −	0.56 (0.53, 0.59)***	_—————_		0.66 (0.62, 0.70)***
Occupation of women	Not worked	− − − −	1.00	_—————_		1.00
	Worked	− − − −	0.78 (0.75, 0.82)***	_—————_		0.76 (0.72, 0.79)***
Marital status of the mother	Married	− − − −	1.00	_—————_		1.00
	Not married	− − − −	1.14 (1.09, 1.19)***	_—————_		1.27 (1.21, 1.33)***
Household family size	1–4	− − − −	1.00	_—————_		1.00
	5–10	− − − −	0.92 (0.87,0.96)	_—————_		0.97 (0.93,1.01)
	> 11	− − − −	1.01 (0.94, 1.08)	_—————_		0.95 (0.87, 1.03)
Media exposure	No	− − − −	1.00	_—————_		1.00
	Yes	− − − −	0.65 (0.62, 0.68)***	_—————_		0.69 (0.66, 0.72)***
Wealth index	Poorest	− − − −	1.00	_—————_		1.00
	Poorer	− − − −	0.89 (0.84, 0.95)*	_———–_		0.88 (0.83, 0.94)*
	Middle	− − − −	0.77 (0.72, 0.82)***	_—————_		0.77 (0.72, 0.82)***
	Richer	− − − −	0.63 (0.59, 0.67)***	_—————_		0.65 (0.61, 0.69)***
	Richest	− − − −	0.43 (0.39, 0.46)***	_—————_		0.46 (0.43, 0.50)***
Birth order	≤3	− − − −	1.00	_—————_		1.00
	> 3	− − − −	1.05 (0.98, 1.12)	_—————_		1.06 (0.99, 1.13)
Sex of child	Male	− − − −	1.00	_—————_		1.00
	Female	− − − −	0.98 (0.94, 1.02)	_—————_		0.98 (0.95, 1.02)
Age of child	6–8	− − − −	1.00	_—————_		1.00
	9–11	− − − −	0.50 (0.47, 0.54)**	_———–_		0.51 (0.47, 0.54)**
	12–23	− − − −	0.32 (0.29, 0.33)***	_———–_		0.31 (0.29, 0.33)***
Plurality of birth	Single	− − − −	1.00	_—————_		1.00
	Multiple	− − − −	0.79 (0.71, 0.88)**	_—————_		0.77 (0.69, 0.87)***
Current breastfeeding	No	− − − −	1.00	_—————_		**1.00**
	Yes	− − − −	1.58 (1.52, 1.66)**	_—————_		1.53 (1.46, 1.60)***
Unplanned pregnancy	No	− − − −	1.00	_—————_		1.00
	Yes	− − − −	1.04 (0.99, 1.09)	_—————_		1.08 (0.99, 1.10)
Place of delivery	Home	− − − −	1.00	_—————_		1.00
	Health institution	− − − −	0.87 (0.83, 0.92)	_———–_		0.87 (0.83, 0.91)**
ANC visit	No ANC visit	− − − −	1.00	_—————_		1.00
	At least one visit	− − − −	0.74 (0.69, 0.78)***		**_—————_**	0.74 (0.69, 0.78)***
Community level variables						
Residence	Urban	− − − −	**_—————_**	1.00		1.00
	Rural	− − − −	**_—————_**	2.27(2.18, 2.36)***		1.29 (1.23, 1.36)***
Region in SSA	Central Africa	− − − −	**_—————_**	1.00		1.00
	East Africa	− − − −	**_—————_**	0.54 (0.50, 0.57)***		0.59 (0.55, 0.63)***
	West Africa	− − − −	**_—————_**	0.51 (0.75, 0.86)***		0.82 (0.76, 0.88)**
	South Africa	− − − −	**_—————_**	0.69 (0.61, 0.77)***		0.83 (0.72, 0.96)**
Country income level	Lower	− − − −	**_—————_**	1.00		1.00
	Lower middle	− − − −	**_—————_**	0.75 (0.72, 0.78)***		0.79 (0.75, 0.83)***
	Upper middle	− − − −	**_—————_**	0.49(0.45, 0.54)***		0.42(0.38, 0.46)***
Survey year	2010–2012	− − − −	**_—————_**	1.00		1.00
	2013–2015	− − − −		0.78 (0.73, 0.82)***		0.95 (0.89, 1.01)
	2016–2019	− − − −		0.73 (0.69, 0.77)**		0.93 (0.89, 1.00)
Community media usage	Low	− − − −	**_—————_**	1.00		1.00
	High	− − − −	**_—————_**	0.81(0.77, 0.85)***		0.99 (0.95, 1.04)
Community-women education	Low	− − − −	**_—————_**	1.00		1.00
	High	− − − −	**_—————_**	0.84 (0.80, 0.88)*		0.95 (0.91, 1.00)
Random effect		− − − −				
	Variance	0.58	0.56	0.57		0.50
	ICC	0.16	0.14	0.15		0.13
	MOR	2.05	2.03	2.05		1.95
	PCV	Reff	0.04	0.02		0.14
Model comparison						
	Log likelihood	−42,188	−36,586	−40,690		−36,279
	Deviance	84,376	73,172	81,380		72,558
	Mean VIF	—	1.59	2.18		1.96

*P-value < 0.05, **P value < 0.01, ***P value < 0.001.

*AOR, adjusted odds ratio; CI, confidence interval; ICC, intercluster correlation coefficient; MOR, median odds ratio; PCV, proportional change in variance; VIF, variance inflation factors.*

*With sample size = 77,887 children aged 6–23 months using multilevel binary logistic regression model.*

Based on the multivariable analysis of the final selected model (model III) results, the chances of having primary and above primary educated women were 8 and 34% less to have inadequate minimum DD intake of their children than women with no formal education (AOR = 0.92; 95% CI; 0.86–0.96) and (AOR = 0.66; 95% CI; 0.62–0.70), respectively. The odds of having children with inadequate minimum DD were 24% reduced among women who were working than those who were not working (AOR = 0.76; 95% CI; 0.72–0.79). Women who were not married were 1.27 times more likely to have children with inadequate minimum DD as compared to those who were married (AOR = 1.27; 95% CI; 1.21–1.33).

The odds of having children with inadequate minimum DD intake were 31% less likely among households, which have exposure to at least one media (radio, TV, or newspaper) than those not exposed (AOR = 0.69; 95% CI; 0.66–0.72). When we consider from poorer to the richest households, inadequate minimum DD intake among children decreased by 12% (AOR = 0.88; 95% CI; 0.83–0.94), 23% (AOR = 0.77; 95% CI; 0.72–0.82), 35% (AOR = 0.65; 95% CI; 1.61–0.69), and 56% (AOR = 0.46; 95% CI; 0.43–0.50) for poorer, middle, rich, and richest households, respectively. Moreover, children from lower middle-income and higher middle-income level countries are also less likely 21 and 58% to have inadequate minimum DD than a child from lower-income level countries (AOR = 0.79; 95% CI; 0.75–0.83) and (AOR = 0.42; 95% CI; 0.38–0.46), respectively.

Children aged 12–23 months were 0.69% less likely to have inadequate minimum DD as compared to a child with 6–8 months of age (AOR = 0.31; 95% CI; 0.29–0.33). Multiple births were 23% less likely to have inadequate minimum DD than those who were singleton (AOR = 0.77; 95% CI; 0.69–0.87). The odds of having inadequate minimum DD among children who are currently breastfed were 1.53 times higher than those who are non-breastfed (AOR = 1.53; 95% CI; 1.46–1.60).

The odds of having inadequate minimum DD of the child were 26 and 13% less likely among mothers who have at least one ANC follow-up and delivery at a health institution (AOR = 0.74; 95% CI; 0.69–0.78) and (AOR = 0.87; 95% CI; 0.83–0.91), respectively.

Children who live in rural residences were 1.29 times more likely to have inadequate minimum DD access than those who live in urban areas (AOR = 1.29; 95% CI; 1.23–1.36). Children who live in the East Africa region were also 41% times less likely to have inadequate minimum DD as compared to those who live in Central Africa (AOR = 0.59; 95% CI; 0.55–0.63) (Table 6).

### Socioeconomic Inequalities of Inadequate Minimum DD Intake

**FIGURE 3:**
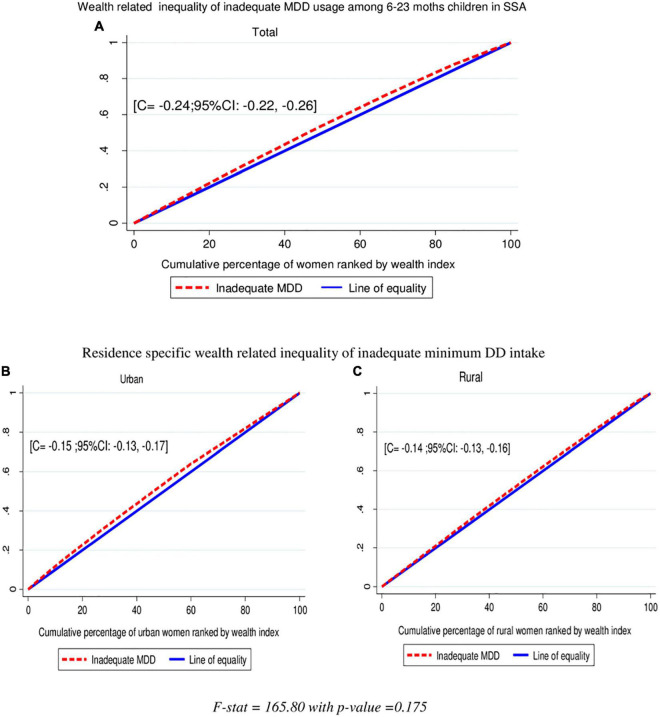
Wealth-related inequalities of inadequate minimum DD intake total **(A)**, urban **(B)**, and rural **(C)** among children aged 6–23 months in SSA.

In this study, the overall wag staff normalized concentration index (C) analyses of the wealth-related inequality of inadequate minimum DD intake showed the pro-poor distribution with (C = −0.24; 95% CI: −0.22, −0.0.26). This shows that inadequate minimum DD intake among children aged 6–23 months was disproportionately concentrated in the poor groups (pro-poor). The concentration index is twice the area between the concentration curve and the diagonal line. Then, when multiplying the C by 75, (−0.24 × 0.75) = 0.18 showed that 18% of the inadequate minimum DD intake would need to be (linearly) redistributed from the richer half to the poorer half of the population to arrive at a distribution with an index value of zero (perfect equality). Even though the wealth-related inequalities of inadequate MDD intake are high among urban residents than rural, it is not statistically significant (C = −0.24; 95% CI: −0.22, −0.0.26) with a *p*-value = 0.175. The finding from the indices is in agreement with the results of the concentration curves which showed that the concentration graph of inadequate minimum DD usage was above the line of equality, which indicated that the distribution of inadequate minimum DD used by children was concentrated in poor households (pro-poor distribution) (Figure 3).

## Discussion

Inadequate IYCF practices are a major problem both globally and in developing countries, and are major determinants of undernutrition, optimal growth, and development, especially in the first 2 years of life (15). Identifying and reducing preventable determinants of malnutrition is a critical step toward improving children’s overall health and well-being (45). This study aimed to assess the pooled estimates of inadequate MDD intake and associated factors among children aged 6–23 months in SSA. Based on this, more than three-fourths, 76.53% (95% CI: 73.37–79.70), of children aged 6–23 months in the SSA countries did not have access to adequate MDD. This is in line with the research conducted in India (77%) (19) and Myanmar (75.4%) (46). But our study indicated that this percentage is higher than studies conducted in Bangladesh (58.1%) (47), Nepal (53.5%) (17), Indonesia (46.7%) (20), Sri Lanka (29%) (21), and a study in two South-East Asian countries, namely, Cambodia (52.3%) and Indonesia (41.8%) (46), which shows that children aged 6–23 months have inadequate access to MDD.

The discrepancy might be due to differences in food cultures (10, 27, 46). This is explained as some societies are agricultural dominant in child feeding, whereas others depend on animal products (46). Geographical variation, population growth, and socioeconomic status of the countries might have also contributed (10, 27, 48). Studies showed that SSA children from early infancy and up to the second year of life have growth problems (49). Governmental actions toward the application of national nutritional programs and addressing cultural beliefs around complementary feeding are also crucial (50). Food availability and accessibility, such as children in agrarian-dominant and city dwellers and from upper middle-income countries were more likely to take adequate MDD (9, 26, 28). For instance, Burkina Faso is among the poorest countries in the world, and almost all children (94.40%) did not access adequate MDD, whereas South Africa was an upper middle-income country, and a half (50.5%) of the children do not have access to nutritional food.

This study also demonstrates that sociodemographic and health factors are associated with MDD in SSA. The current study found that as the age of children increases, the odds of having inadequate minimum DD decreases, which implies that the practice of adequate minimum DD significantly increases as the child’s age increases. This is similar to studies conducted in Indonesia (9), Bangladesh (47), Malawi (16), and Ethiopia (14, 28).

This could be because of the fact that mothers may perceive that younger children have the poor ability of the intestine to digest certain foods like bananas, eggs, pumpkin, carrots, green vegetables, and meat (51). So, regarding the late introduction of complementary feeding and when they start complementary feeding on time, they included only milk or cereal products (24, 28). On the other side, the loss of appetite during teething and weight loss due to increased infections could explain changes in feeding practices among children aged 6–8 months (52).

This study showed that, as the educational status of women improves, so do their chances of having children who did not access MDD. This is supported by a study conducted in Indonesia (9), Bangladesh (47), South Asia (22), India (27), Tanzania (25), Ghana (53), and Ethiopia (14, 54–56). Mothers’ knowledge of child feeding was positively associated with MDD in child-feeding practice (10, 12, 54). As educated women are more likely to have access to quality health services and messages, they may more easily comprehend and translate that information into practice (16, 51).

In this study, women who are currently not married were more likely to have children with inadequate access to MDD as compared to those who were married. This is the same with research conducted in Indonesia (9), India (27), and Ethiopia (57). This might be due to that, as currently unmarried women are single, widowed, or divorced, they have no support from husbands and less support from families or communities, which causes poor infant-feeding practices (9, 58).

In this study, children who have employed mothers were less likely to get inadequate MDD. This is supported by a study conducted in West African countries (24), Malawi (16), Tanzania (25), and Ethiopia (8, 28). The possible explanation might be that women with formal employment may be in a better socioeconomic position to achieve food security, which in turn may influence their complementary feeding practices (16). Employed mother mostly has a higher education status, which eventually leads the household decision-making process to purchase and feed the adequate type of food that is necessary for their child (25).

Children whose mothers have access to the media had a significantly lower chance of inadequate MDD. This is similar to a study conducted in India (27), South Asia (22), West African countries (24), and Malawi (16). This could be attributed to the fact that media, which is generally regarded as a reliable source of health and nutrition information, provides knowledge and affects behavioral change in the mothers (16).

In our study, children who are currently breastfed are more likely to have inadequate access to MDD than those who are non-breastfed, which is contradictory with other studies (26). This might be that, if the mother has breastfed, they are considering their child access to enough food without thinking of giving them complimentary food (24).

According to this study, if mothers have ANC visits during their pregnancy and have an institutional delivery, then they have a lower chance of having inadequate MDD. This is in line with a study conducted in India (27), South Asia (22), and West African countries (24). This might be due to effective nutrition education and counseling (often provided during ANC visits) that might contribute to dietary diversity (27).

In this study, children who lived in rural residences were more likely to have inadequate MDD. This is reinforced by a study conducted in Indonesia (9), South Asia (22), and West African countries (24). The reason for having a higher chance of using adequate MDD in the urban residence might be the result of the cumulative effect of a series of more favorable conditions, including better socioeconomic and educational conditions, in turn leading to better caring practices for children and their mothers (59, 60).

In this study, we found that as the wealth status of the households increases from poorer to richest, the chance of having children who use inadequate MDD significantly decreases. Children from lower-middle and higher middle-income level countries also have less chance to have inadequate MDD as compared to lower-income levels. This is in line with a study conducted in India (27, 61, 62), Bangladesh (47), South Asia (22), Tanzania (25), and Ethiopia (12, 55, 56), but other studies in Ethiopia showed that no association with it (10, 14). This difference might be due to the small sample size and covers the small district of the latter two studies. But it is known that children from a family of higher income can feed diversified foods, as their families could be more likely to afford to have diversified foods as compared to children from a low household income (59).

The main strength of this study was the use of the weighted nationally representative data of each SSA country with a large sample, which makes it representative at sub-Saharan and regional levels. Therefore, it has appropriate statistical power that can be generalized to the estimate inadequate MDD intake in the study setting for all children aged 6–23 months during the study period. Another strength of this study was that estimating the pooled estimate of inadequate MDD intake in SSA and sub-regions will give invaluable information for region-specific intervention. Cross-sectional data were collected at a different time point by self-reported interviews, which would be prone to recall and social desirability bias. The drawback of the secondary nature of data was inevitable. The heterogeneity of the pooled estimate of inadequate MDD intake was not managed by further analysis.

## Conclusion and Recommendation

The magnitude of inadequate MDD intake among children aged 6–23 months in SSA is relatively high. Individual-level factors such as secondary and above maternal education, having an employed mother, having exposure to media, richest wealth, and having health institution delivery have a significant association with inadequate MDD, and among community-level variables, living in an upper middle-income country, living in East Africa region, and living in a rural residence have a significant association with inadequate MDD intake among children aged 6–23 months in SSA.

To decrease inadequate MDD intake among children aged 6–23 months in SSA, policy makers in nutritional projects and other stakeholders should work as an integrated approach with other sectors, and give prior attention to modifiable socioeconomic factors such as promoting women’s education and employment as well as breastfeeding behavior, and increasing wealth status and media exposure of the household.

Interventions to improve inadequate MDD intake should not only be implemented on factors at the individual level but also be tailored to the community context. SSA needs equity-focused interventions to curb the high magnitude and inequalities in inadequate MDD.

## Data Availability Statement

Publicly available datasets were analyzed in this study. This data can be found here: https://dhsprogram.com/data/dataset_admin/login_main.cfm?CFID=39421058&CFTOKEN=a8b8a36f1fb27230-E89DAEA4-D47B-719A-9AD1F13D7D93EF8A.

## Ethics Statement

All methods were carried out following relevant guidelines and regulations of the Institutional Review Boards (IRB) of the University of Gondar (UOG) and the Demographic and Health Surveys (DHS) program. Ethical clearance was obtained from the Institutional Review Boards (IRB) of the University of Gondar (UOG) College of Medicine and Health Sciences (Ref No/IPH/1446/2013). Informed consent was waived from the International Review Board of Demographic and Health Surveys (DHS) program data archivists after the consent manuscript was submitted to DHS Program/ICF International Inc., a letter of permission to download the dataset for this study. The dataset was not shared or passed on to other bodies and has maintained its confidentiality. The study is not an experimental study.

## Author Contributions

DB, SF, WN, ES, and DA contributed to the conception of the work, design of the work, acquisition of data, analysis, and interpretation of data. RT, FA, TA, HE, EF, and DB contributed to the data curation, drafting of the article, revising it critically for intellectual content, validation, and final approval of the version to be published. All authors read and approved the final manuscript.

## Conflict of Interest

The authors declare that the research was conducted in the absence of any commercial or financial relationships that could be construed as a potential conflict of interest.

## Publisher’s Note

All claims expressed in this article are solely those of the authors and do not necessarily represent those of their affiliated organizations, or those of the publisher, the editors and the reviewers. Any product that may be evaluated in this article, or claim that may be made by its manufacturer, is not guaranteed or endorsed by the publisher.

## Abbreviations

AOR: Adjusted Odds Ratio
CI: Confidence Interval
CSA: Central Statistical Agency
DD: Dietary Diversity
DHS: Ethiopian Demographic and Health Survey
KR: Kids Record
MOH: Ministry Of Health
MDD: Minimum dietary diversity.
